# A Case Report of Spontaneous Pneumomediastinum Following Chest Massage: A Rare Clinical Presentation

**DOI:** 10.5811/cpcem.61653

**Published:** 2026-08-31

**Authors:** H Naveed UI Hassan, B G Kowsthubha, Amaravathi Udhayakumar

**Affiliations:** * Jawaharlal Institute of Postgraduate Medical Education and Research, Puducherry, India; † Jawaharlal Institute of Postgraduate Medical Education and Research, Department of Emergency Medicine, Puducherry, India

**Keywords:** chest massage, spontaneous pneumomediastinum, Macklin effect, mediastinitis, mediastinal abscess

## Abstract

**Introduction:**

Spontaneous pneumomediastinum is a rare condition characterized by the presence of air in the mediastinum without preceding trauma. It is often triggered by minor events such as coughing, physical exertion, or traditional therapies.

**Case Report:**

A 52-year-old male with a history of pulmonary tuberculosis developed sudden left-sided chest pain and swelling following traditional chest massage therapy. Examination revealed subcutaneous emphysema with palpable crepitus. Imaging showed characteristic signs of pneumomediastinum with bilateral lung opacities. As a complication of pneumomediastinum the patient developed mediastinitis and a mediastinal abscess, requiring open thoracotomy and drainage. He recovered with multidisciplinary care.

**Conclusion:**

Early recognition of spontaneous pneumomediastinum in emergency settings is crucial, especially when chest pain is accompanied by subcutaneous emphysema, to prevent serious complications.

## INTRODUCTION

Spontaneous pneumomediastinum, also known as Macklin or Hamman syndrome, is an uncommon clinical entity with an incidence of approximately one in 30,000 emergency visits.^1^ It involves the presence of free air in the mediastinum without antecedent trauma, predominantly affecting young males. The pathogenesis involves alveolar rupture caused by a sudden increase in intra-alveolar pressure, with air dissecting along bronchovascular sheaths—the Macklin effect.^2^,^3^ Common triggers include vigorous coughing, strenuous physical activity, labor, vomiting, and the Valsalva maneuver.^1^ Underlying pulmonary diseases, such as asthma and tuberculosis, may predispose individuals to spontaneous pneumomediastinum.

Chest pain is the most frequent symptom, often accompanied by dyspnea, cough, or neck swelling. Although usually benign, the condition can lead to serious complications such as mediastinitis and myocardial injury if not promptly diagnosed, which can be life-threatening. This case is distinctive due to the occurrence of spontaneous pneumomediastinum in a middle-aged man precipitated by traditional massage therapy—an unusual trigger. The patient experienced mediastinitis, mediastinal abscess, and non-ischemic myocardial injury, highlighting diagnostic and management challenges.

## CASE REPORT

A 52-year-old male presented to the emergency department (ED) with continuous dull aching left-sided chest pain and chest wall swelling for four days. Symptoms began after receiving traditional chest massage therapy. He denied trauma, respiratory symptoms, fever, or systemic complaints. His past history included pulmonary tuberculosis treated with a six-month course of antitubercular therapy three years prior and type two diabetes mellitus managed with oral hypoglycaemic agents. He was a former smoker with a history of alcoholism who had abstained for the prior five years. Family and psychosocial histories were unremarkable.

On physical examination, vital signs were normal except for elevated blood glucose. Oxygen saturation was 98% on room air and respiratory rate 20 breaths per minute. Local inspection revealed an 8 x 6 cm area of edema on the left anterior chest wall extending from the clavicle to the nipple, with palpable crepitus and tenderness. Cardiovascular and respiratory examinations were unremarkable. Electrocardiogram (ECG) showed normal sinus rhythm, complete right bundle branch block, and right axis deviation without dynamic changes on serial ECG.

*CPC-EM Capsule*

What do we already know about this clinical entity?

*Spontaneous pneumomediastinum is a rare, usually benign condition caused by alveolar rupture after raised intrathoracic pressure.*

What makes this presentation of disease reportable?

*Traditional chest massage triggered pneumomediastinum complicated by mediastinitis, mediastinal abscess, and myocardial injury.*

What is the major learning point?

*Chest pain with subcutaneous emphysema should prompt evaluation for pneumomediastinum and monitoring for cardiac complications.*

How might this improve emergency medicine practice?

*Early chest radiograph and electrocardiogram-troponin assessment in suspected pneumomediastinum may prevent delayed, life-threatening complications.*

Chest radiograph revealed subcutaneous emphysema over the left pectoralis muscle with the characteristic ginkgo leaf sign and fibrotic bands in the right middle lung zone (Image 1). High-resolution computed tomography (CT) of the thorax confirmed left-sided pneumomediastinum extending into the sternoclavicular joint, manubrium sterni, and pectoralis muscle (Image 2). Patchy consolidations and centrilobular nodules were present bilaterally.

In the ED, the patient was started on analgesics and empirical intravenous (IV) antibiotics. He was admitted under pulmonary medicine and treated with IV clindamycin (600 mg thrice daily), analgesics, and insulin for glycemic control. Multiple skin incisions were done over the subcutaneous emphysema in the left chest wall by the cardiovascular and thoracic surgery team to relieve the subcutaneous emphysema.

On hospital day 3, the patient developed sinus tachycardia with persistent axis deviation. Troponin I was elevated with a value of 93 ng/L (reference range, < 15 ng/L) without significant serial change, suggestive of nonischemic myocardial injury. On hospital day 7, he developed mediastinitis with a mediastinal abscess requiring intubation and open thoracotomy for drainage.

Intraoperative cultures were sterile. The patient was extubated the following day, transitioned to oral antibiotics, and improved clinically. He was discharged in stable condition.

## DISCUSSION

Spontaneous pneumomediastinum occurs due to alveolar rupture caused by a sudden rise in intrathoracic pressure, allowing air to track along the bronchovascular sheaths into the mediastinum—a phenomenon known as the Macklin effect.^2^,^3^ Chest pain and subcutaneous emphysema are hallmark signs, although symptoms may vary. The ginkgo leaf sign on chest radiograph and high-resolution CT confirmation are key diagnostic modalities.^5^.^6^ The ginkgo leaf sign is a distinctive radiographic finding in spontaneous pneumomediastinum, seen as a fan-shaped lucency created by air dissecting between the pectoralis major muscle and the chest wall. This appearance resembles the shape of a ginkgo biloba leaf and serves as a useful clue on chest radiograph to identify subcutaneous emphysema and underlying pneumomediastinum.^5^ The Hamman sign (a crunching sound synchronous with the heartbeat) may aid in diagnosis but was absent in this case. The patient was thought to have pneumothorax or esophageal rupture, later diagnosed to have spontaneous pneumomediastinum.

While most cases resolve with conservative treatment—oxygen, analgesia, and observation—complications such as mediastinitis, mediastinal abscess, and myocardial injury may occur, particularly in those with pre-existing pulmonary disease.^5^ Empirical antibiotics are typically reserved for suspected infections or complications.^8^ Asymptomatic patients with stable vitals and no high-risk features can be observed and safely discharged with a follow-up date.

Our patient’s spontaneous pneumomediastinum was precipitated by traditional chest massage, likely increasing intrathoracic pressure and causing alveolar rupture. Prior pulmonary tuberculosis may have weakened the lung parenchyma, enhancing susceptibility. The patient received multiple skin incisions for subcutaneous emphysema for decompression, although the literature supports a single incision over the emphysema.^9^ Despite early antibiotics, he developed mediastinitis and abscess requiring surgical drainage, a rare but severe complication. The raised troponin I level in our patient, without significant serial rise or fall, likely reflected nonischemic myocardial injury secondary to inflammatory mediastinal involvement rather than acute coronary syndrome. The mediastinitis and local inflammation can cause myocardial irritation or myocarditis-like injury, leading to myocardial cell damage and troponin release.^7^

Spontaneous pneumomediastinum associated with elevated troponin levels should prompt consideration of myocarditis or inflammatory cardiac injury secondary to mediastinal inflammation. Echocardiography or cardiac magnetic resonance imaging can aid in further characterization, although such investigations were deferred in this case due to clinical improvement.

The development of mediastinitis in spontaneous pneumomediastinum is extremely rare but can be fatal if missed. Possible mechanisms include secondary infection of mediastinal air collections or translocation of skin flora during subcutaneous dissection. Persistence of chest pain despite adequate analgesia raised concerns for alternative causes and underlying complications, our case underscores the need for close clinical monitoring and early imaging follow-up even in seemingly benign spontaneous pneumomediastinum.

Atypical triggers such as chest massage should be recognized by clinicians, especially in patients with compromised lung architecture. A multidisciplinary approach involving emergency medicine, pulmonology, and thoracic surgery is vital in complex cases. This case illustrates the importance of prompt diagnosis and multidisciplinary management.

## CONCLUSION

Spontaneous pneumomediastinum is a rare yet important cause of chest pain that may follow uncommon triggers such as traditional chest massage. Although often benign, it can lead to serious complications such as mediastinitis and myocardial injury. The presence of spontaneous, symptomatic subcutaneous emphysema warrants emergency physicians to maintain a high index of suspicion and to proceed for advanced imaging. Timely imaging and multidisciplinary intervention are essential for optimal outcomes.

## Figures and Tables

**Image 1:**
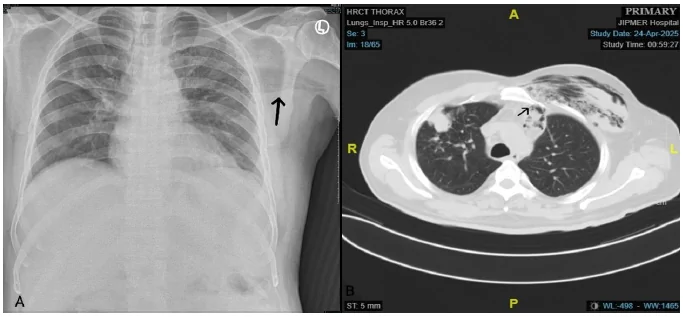
A) chest radiograph in posteroanterior view showing the left pectoralis muscle air collection (arrow), ginko leaf sign, and right middle zone fibrotic bands. B) High-resolution computed tomography of the chest in axial section showing pneumomediastinum predominantly over the left side (arrow), with consolidation in the right lung and air collection in the sternoclavicular joint and left pectoralis muscle.

**Image 2:**
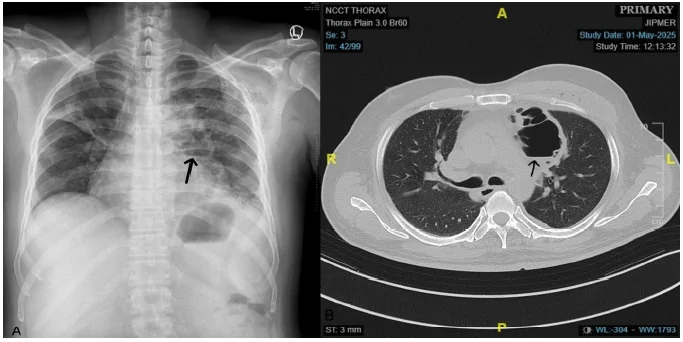
A) The chest radiograph in posteroanterior view now demonstrates air-fluid level at the left side of the pneumomediastinum (arrow), suggesting mediastinal abscess and right middle zone fibrotic bands. B) Repeat high-resolution computed tomography in axial view now shows left-sided mediastinal collection with air-fluid level within it.

## References

[1] Macia I, Moya J, Ramos R (2007). Spontaneous pneumomediastinum: 41 cases. Eur J Cardiothorac Surg.

[2] Newcomb AE, Clarke CP (2005). Spontaneous pneumomediastinum: a benign curiosity or a significant problem?. Chest.

[3] Macklin CC (1939). Transport of air along sheaths of pulmonic blood vessels from alveoli to mediastinum: clinical implications. Arch Intern Med.

[4] Koullias GJ, Korkolis DP, Wang XJ (2004). Current assessment and management of spontaneous pneumomediastinum: experience in 24 adult patients. Eur J Cardiothorac Surg.

[5] Murayama S, Gibo S (2014). Spontaneous pneumomediastinum and Macklin effect: overview and appearance on computed tomography. World J Radiol.

[6] Maunder RJ, Pierson DJ, Hudson LD (1984). Subcutaneous and mediastinal emphysema. Pathophysiology, diagnosis, and management. Arch Intern Med.

[7] Chiang HT, Tsai CT, Hsieh MJ (2018). Non-ischemic myocardial injury secondary to mediastinal inflammation in spontaneous pneumomediastinum: a diagnostic challenge. J Emerg Med.

[8] Gologorsky R, Oklu R (2020). Spontaneous pneumomediastinum: pathophysiology and management. Cardiol Clin.

[9] Aghajanzadeh M, Dehnadi A, Ebrahimi H (2015). Classification and management of subcutaneous emphysema: a 10-year experience. Indian J Surg.

